# Supplementary material for: Common Variants in IL-20 Gene are Associated with Subclinical Atherosclerosis, Cardiovascular Risk Factors and IL-20 Levels in the Cohort of the Genetics of Atherosclerotic Disease (GEA) Mexican Study
Source: Biomolecules. 2020 Jan 3;10(1):75. doi: 10.3390/biom10010075 (PMC7022804; doi:10.3390/biom10010075)
Supplement: Supplementary File 1 [file biomolecules-10-00075-s001.pdf]

Table 1. Supplemental Material. Association between *IL-20* gene polymorphisms and subclinical atherosclerosis.

			Women					Men				
**SNP**	**Model**	**Genotypes and Alleles**	**SA**	**Control**	**P**	**OR**	**95% CI**	**SA**	**Control**	**P**	**OR**	**95% CI**
			**n**	**n**				**n**	**n**			
rs1400986		CC	53	202	0.0009	0.43		124	134	0.087	0.67	0.45-1.00
		CT	21	187				66	106			
		TT	1	26				9	17			
		C	127	591	0.0002	0.44	0.28-0.71	314	374	0.036	0.71	0.52-0.97
		T	23	239				84	140			
	codominant1	CC	53	202	0.002	0.42	0.24-0.73	124	134	0.048	0.67	0.45-0.99
		CT	21	187				66	106			
	codominant2	CC	53	202	0.036	0.14	0.01-1.10	124	134	0.219	0.57	0.24-1.33
		TT	1	26				9	17			
	dominant	CC	53	202	0.0004	0.39	0.23-0.67	124	134	0.029	0.66	0.45-0.96
		CT+TT	22	213				75	123			
	recessive	CC+CT	74	389	0.046	0.2	0.03-1.51	190	240	0.33	0.67	0.29-1.53
		TT	1	26				7	17			
	additive	-	-	-	0.0002	0.42	0.26-0.68	-	-	0.03	0.71	0.52-0.97
rs1518108		CC	25	112				54	69	0.089	1.17	0.75-1.82
		CT	33	220	0.34	0.67		106	116			
		TT	17	83				39	72			
		C	83	444	0.596	0.89	0.63-1.26	214	254	0.204	0.84	0.64-1.09
		T	67	386				184	260			
	codominant1	CC	25	112	0.181	0.67	0.38-1.18	54	69	0.501	1.16	0.74-1.81
		CT	33	220				106	116			
	codominant2	CC	25	112	0.864	0.91	0.46-1.80	54	69	0.183	0.69	0.40-1.17
		TT	17	83				39	72			
	dominant	CC	25	25	0.27	0.74	0.44-1.25	54	69	0.95	0.99	0.65-1.50
		CT+TT	50	303				145	188			
	recessive	CC+CT	58	332	0.6	1.17	0.65-2.12	160	185	0.036	0.63	0.40-0.98
		TT	17	83				39	72			
	additive	-	-	-	0.67	0.93	0.65-1.32	-	-	0.2	0.84	0.65-1.09

Table shows the models with significant association. SA: Subclinical atherosclerosis.
